# Emerging Contributions of Solid-State NMR Spectroscopy to Chromatin Structural Biology

**DOI:** 10.3389/fmolb.2021.741581

**Published:** 2021-10-11

**Authors:** Bryce E. Ackermann, Galia T. Debelouchina

**Affiliations:** Department of Chemistry and Biochemistry, University of California, San Diego, La Jolla, CA, United States

**Keywords:** gene regulation, magic angle spinning, chemical biology, nucleosome dynamics, histone dynamics

## Abstract

The eukaryotic genome is packaged into chromatin, a polymer of DNA and histone proteins that regulates gene expression and the spatial organization of nuclear content. The repetitive character of chromatin is diversified into rich layers of complexity that encompass DNA sequence, histone variants and post-translational modifications. Subtle molecular changes in these variables can often lead to global chromatin rearrangements that dictate entire gene programs with far reaching implications for development and disease. Decades of structural biology advances have revealed the complex relationship between chromatin structure, dynamics, interactions, and gene expression. Here, we focus on the emerging contributions of magic-angle spinning solid-state nuclear magnetic resonance spectroscopy (MAS NMR), a relative newcomer on the chromatin structural biology stage. Unique among structural biology techniques, MAS NMR is ideally suited to provide atomic level information regarding both the rigid and dynamic components of this complex and heterogenous biological polymer. In this review, we highlight the advantages MAS NMR can offer to chromatin structural biologists, discuss sample preparation strategies for structural analysis, summarize recent MAS NMR studies of chromatin structure and dynamics, and close by discussing how MAS NMR can be combined with state-of-the-art chemical biology tools to reconstitute and dissect complex chromatin environments.

## Introduction

**FIGURE 1:**
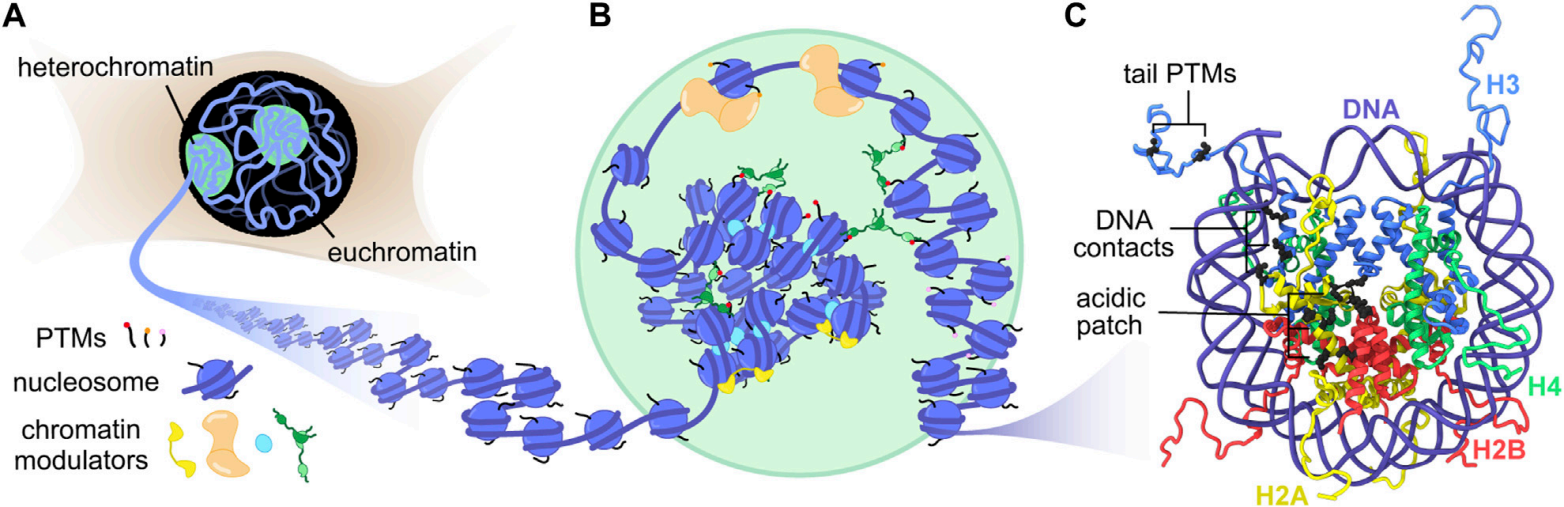
Genome organization from the nucleus to the nucleosome. **(A)** The nucleus contains two distinct chromatin states, heterochromatin and euchromatin. Compact heterochromatin compartments may form by phase separation. **(B)** Chromatin fibers in different states contain distinct PTM signatures and interact with specific chromatin modulators. **(C)** The structure of the nucleosome with highlighted regions of interest for MAS NMR studies (PDB:1KX5) (Davey et al., 2002).

In the nuclei of eukaryotic cells, approximately 2 m of DNA must be packaged and organized for efficient gene regulation and DNA replication (Figure 1A). On a global level, this is achieved by separation into active gene regions called euchromatin and silent gene compartments known as heterochromatin. A fundamental molecular building block for such organization is the nucleosome, a complex of histone proteins (2 each of H2A, H2B, H3, and H4) assembled into an octamer and wrapped by ∼147 base pairs of DNA. These nucleosome units repeat along the length of each chromosome to generate a “‘beads-on-a-string” polymer called chromatin (Woodcock et al., 1976) (Figure 1B). The interactions between histones and DNA are primarily electrostatic in nature, where the peripheral basic residues of the histone octamer intercalate into the phosphate backbone of wrapped DNA, while the dynamic histone termini (tails) transiently explore exposed DNA (Figure 1C). Since the majority of the genome is bound by histones, any modification of these interactions, however small, can alter genetic outcomes. Post-translational modifications (PTMs), for example, differentially mark histone tails to recruit specific histone readers (Kouzarides, 2007; Prakash and Fournier, 2017) or to initiate DNA unwrapping (Bowman and Poirier, 2015). Macromolecular complexes that recognize such PTMs can further impact chromatin organization by cross-linking nucleosomes that are megabases apart in sequence (Rao et al., 2014; Strom et al., 2021), or by shifting the position of nucleosomes to expose new DNA sites for transcription initiation. Despite continuing progress towards determining the structure of chromatin in cells (Hsieh et al., 2015; Ricci et al., 2015; Nozaki et al., 2017; Ou et al., 2017; Risca et al., 2017; Cai et al., 2018; Xu et al., 2018; Ohno et al., 2019; Otterstrom et al., 2019; Krietenstein et al., 2020; Su et al., 2020), the impressive span of length scales involved, from small chemical modifications in the Ångstrom range to whole chromosome rearrangements on the micrometer scale, creates a tremendous challenge for structural biologists and biophysicists.

Bottom-up approaches using purified components are well suited to systematically probe the interplay between nucleosomes and chromatin structure on the Ångstrom and nanometer scale (Allis and Muir, 2011). The isolation of mononucleosomes from their polymer context enables high-resolution structural biology where the rigid histone core can be studied by X-ray crystallography and cryo-EM, and the histone tails by solution NMR spectroscopy (McGinty et al., 2016; Zhou et al., 2019). Virtually any chromatin and DNA process has now been mapped on the nucleosome, including chromatin remodeling, transcription, histone/DNA modification, gene repression, and DNA repair (Jang and Song, 2019; Zhou et al., 2019; McGinty and Tan, 2021; Min and Liu, 2021). These studies have highlighted several cornerstones in nucleosome recognition and modification. The H2A-H2B acidic patch, a lone cluster of glutamate and aspartate residues on the nucleosome surface, serves as a landing pad to anchor chromatin modulators as well as nearby nucleosomes (Kalashnikova et al., 2013; Chen et al., 2017; McGinty and Tan, 2021) (Figure 1C). The histone tails are dynamic, enabling access to both modification and recognition. PTMs and sequence variation of histones dictate the dynamics of nucleosome sliding and unwrapping. The histone-induced bending of nucleosomal DNA elicits unique recognition motifs for protein interaction.

Building upon these studies, the interactions of many adjacent nucleosomes can be addressed. The chromatin context is important for biomolecular recognition; some chromatin modulator complexes are much larger than a nucleosome and can sense nearby nucleosomes (Yang et al., 2006; He et al., 2020), many architectural proteins are multivalent and can simultaneously interact with several nucleosomes (Machida et al., 2018; Poepsel et al., 2018), and neighboring nucleosomes can stack atop each other, thereby competing with chromatin modulators for binding sites (Bilokapic et al., 2018; Sanulli et al., 2019; Alvarado et al., 2021). The fiber context is also necessary for packaging, as nucleosomes are strung together and densities get closer to those observed in cells (10–100 mg/ml) (Imai et al., 2017; Hancock, 2018; Kim and Guck, 2020), chromatin can undergo phase separation into a highly viscous solid-like material (Strickfaden et al., 2020). Phase separation is a promising model for cellular compartmentalization (and more recently chromatin compaction) that depends on a large number of transient multivalent interactions (Gibson et al., 2019; Sanulli et al., 2019). While this setting better represents the native nucleosome competition and the physical forces of compaction, the heterogeneity and density of such nucleosome polymers become intractable to most structural techniques.

**FIGURE 2:**
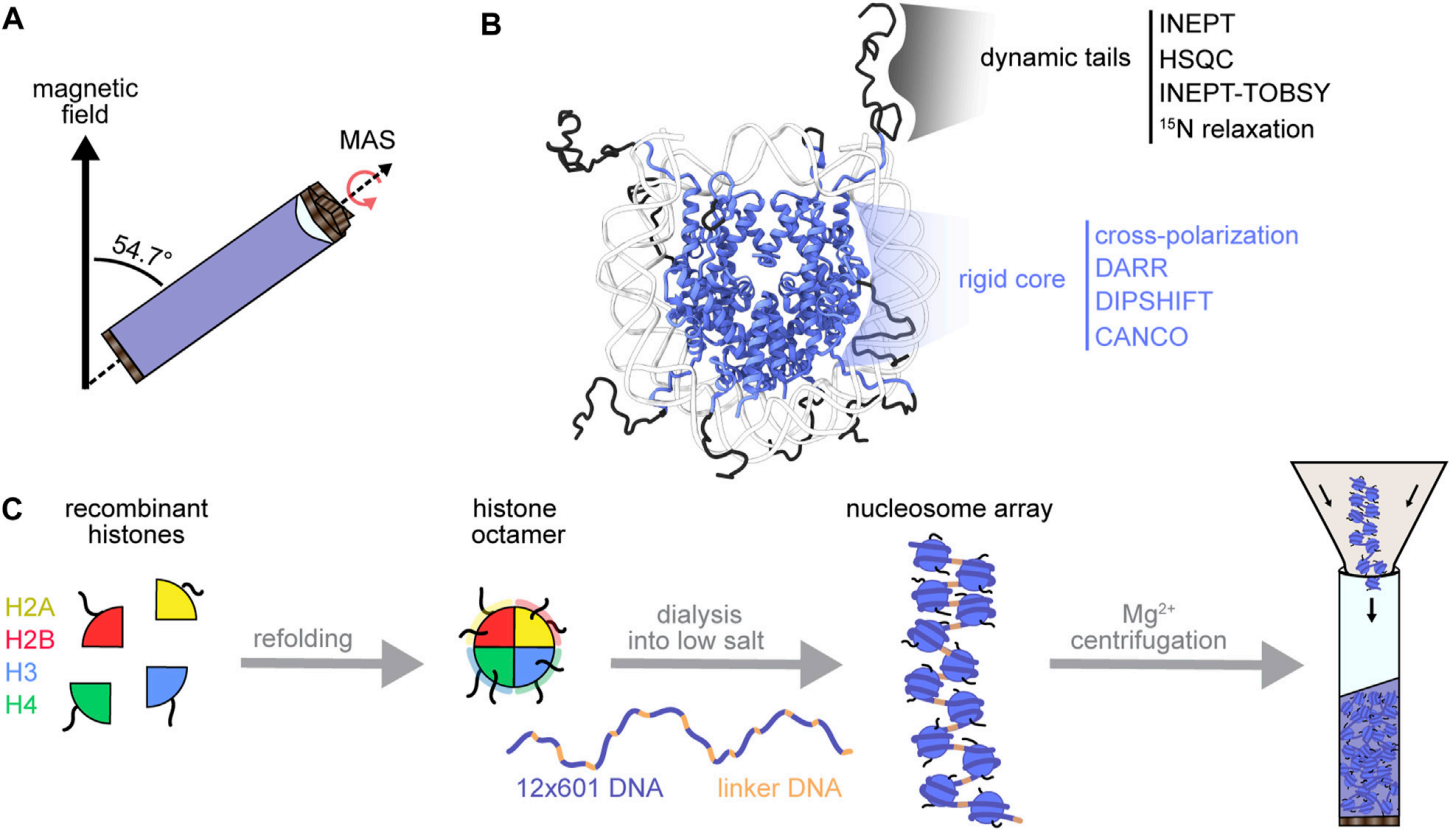
MAS NMR toolbox for chromatin structural biology. **(A)** During MAS NMR, the sample rotor is spun at frequencies between 10 and 100 kHz at the magic angle (54.7° relative to the external magnetic field). **(B)** MAS NMR can probe the dynamic range of the nucleosome with experiments designed to detect either the mobile histone tails or the rigid nucleosome core (PDB:1KX5) (Davey et al., 2002). **(C)** Chromatin reconstitution begins with the formation of histone octamers from recombinant histones, followed by DNA wrapping at low salt. Mg^2+^ can be used to purify arrays and to compact chromatin during rotor packing.

NMR spectroscopy rises to the forefront of techniques uniquely capable of probing atomic structural and dynamic information for complex samples. Solution NMR of proteins benefits from fast molecular rotation to average line-broadening anisotropic interactions. However, larger proteins or protein complexes may tumble too slowly for efficient averaging and may require the tools of solid-state NMR. Magic-angle spinning solid-state NMR spectroscopy (MAS NMR) achieves partial averaging by rapid (tens of kilohertz) rotation of the sample at 54.7° (the magic angle) relative to the external magnetic field (Figure 2A). MAS thereby enables detection of large macromolecules in various material states (amorphous, crystalline, gel-like, liquid) (Quinn and Polenova, 2017; van der Wel, 2018). MAS NMR can detect both slow and fast biomolecular motions by using solution-state (INEPT) experiments that depend on through-bond scalar-couplings (Morris and Freeman, 1979; Andronesi et al., 2005) or by using solid-state cross-polarization (CP) experiments (Hartmann and Hahn, 1962; Pines et al., 1973) that rely on through-space dipolar interactions (Figure 2B). These two experiment types allow for qualitative dynamics-based spectral filtering (Matlahov and van der Wel, 2018), where INEPT selects for nuclei that undergo dynamic isotropic motion, and CP builds up signal for nuclei in rigid networks that maintain strong dipolar couplings. MAS NMR can also resolve distance parameters by recoupling spatially informative anisotropic interactions via pulse sequences matched to the sample spinning rate. These features of MAS NMR suit chromatin, a heterogenous polymer that forms an amorphous solid *in vitro* and *in nucleo* (Strickfaden et al., 2020), and that bears nucleosomes with both rigid histone cores and dynamic histone tails (Fierz and Poirier, 2019). Therefore, MAS NMR can detect the dynamic range of nucleosomes while embedded in the chromatin context. In this review, we will cover the practicalities of sample preparation for MAS NMR, highlight the current applications of MAS NMR to chromatin, and finally outline the next frontier of biologically compelling chromatin design and structural analysis. Readers interested in chromatin applications of other structural techniques such as solution NMR spectroscopy, X-ray crystallography and cryo-EM are referred to several recent comprehensive reviews on these topics (van Emmerik and van Ingen, 2019; Zhou et al., 2019; McGinty and Tan, 2021).

## Chromatin Sample Preparation for MAS NMR

The chromatin fiber, while richly decorated in cells with PTMs, histone variants and chromatin modulators, can be minimally reconstructed using purified DNA and recombinant histones (Figure 2C). Mononucleosomes and nucleosome arrays were first reconstituted by depositing histone octamers onto alpha-satellite DNA and tandem-repeat 5S rDNA, respectively (Simpson et al., 1985; Luger et al., 1997; Fierz and Muir, 2012). The discovery of the high-affinity 601 positioning sequence soon revolutionized chromatin construction by enabling the efficient assembly of highly homogenous chromatin preparations (Lowary and Widom, 1998). The 601 DNA is now favored in most *in vitro* biochemical and structural chromatin studies. Nucleosome arrays have been standardized to include twelve 601 sequence repeats, partitioned by selected lengths of linker DNA (Huynh et al., 2005). The 12-mer DNA, along with a short helper DNA strand that assists later steps, are cloned for bacterial production on a large scale (tens of milligrams). Histone octamers, on the other hand, are formed by refolding a stoichiometric mixture of the four histone proteins, typically produced recombinantly in *E. coli* and thus devoid of PTMs. For NMR studies, the histone of interest is expressed in isotopically enriched media which leads to a selectively labeled histone type within the otherwise NMR-silent nucleosome complex. Finally, the repetitive DNA and helper DNA strands are excised from their parent plasmids and loaded with octamers by the salt dialysis assembly method to produce well-defined and spaced nucleosome arrays (Dorigo et al., 2003; Dyer et al., 2003; Fyodorov and Kadonaga, 2003) (Figure 2C). The helper DNA, which has a weaker affinity for histones, is essential for soaking up the excess histone octamers required to saturate the twelve nucleosome sites in the array. Nucleosome arrays can then be purified from helper DNA by a facile Mg^2+^ precipitation step, in contrast to mononucleosomes which require sucrose gradient or preparative gel purifications to remove leftover DNA. Homotypic and heterotypic post-translationally modified nucleosome arrays can be produced using native chemical ligation, expressed protein ligation, unnatural amino acid incorporation and other chemical approaches (Müller and Muir, 2015; Muller et al., 2016). While these methods typically yield small amounts of modified nucleosome arrays, some can be adapted to the high sample demands of NMR spectroscopy. We will briefly discuss those in the last section of this review.

The development of efficient and reliable protocols for chromatin assembly has led to fundamental insights into chromatin structure. For example, it is now well known that the H4 tail and the H2A/H2B acidic patch are critical for inter-nucleosome stacking (Dorigo et al., 2003; Zhou et al., 2007; Lu et al., 2008) while lysine acetylation on the H4 tail can open up the chromatin fiber and expose DNA (Shogren-Knaak, 2006; Mishra et al., 2016). Chromatin reconstitution was also vital for uncovering the structural continuum of extended 10-nm fibers, folded 30-nm fibers, interdigitated fibers, and irregular globules that depend on Mg^2+^ concentration, DNA linker length, linker histone H1, and array concentration (Baldi et al., 2018; Maeshima et al., 2019; Adhireksan et al., 2020; Brouwer et al., 2021). Despite the detection of structured high-order fibers *in vitro*, recent studies emphasize that interphase chromatin appears to compact irregularly at densities up to 100 mg/ml (Poirier et al., 2009; Hihara et al., 2012; Imai et al., 2017; Cai et al., 2018; Hancock, 2018; Kilic et al., 2018; Audugé et al., 2019). Careful preparation and explicit characterization of chromatin states stands as a crucial step to identify the specialized structures that relate to functions such as gene repression, mitosis, and transcription (Luger et al., 2012).

MAS NMR is well suited to tackle macroscopically heterogenous samples that emulate the irregularity of nuclear chromatin. Non-crystalline samples are routinely packaged for analysis by ultracentrifugation (Bertini et al., 2013; Mandal et al., 2017), where high gravity values generate maximally concentrated sediments of macromolecules while retaining sample hydration and stability for years (Fragai et al., 2013; Wiegand et al., 2020). Sedimentation can be performed in several ways; the sample can be centrifuged inside a tube and then transferred to a rotor, centrifuged directly into a rotor, or sedimented inside the rotor during MAS (Bertini et al., 2013; Mandal et al., 2017). The rate of sedimentation depends on the degree of chromatin compaction (Dorigo et al., 2003) and the process can be sped up by the addition of Mg^2+^ ions. Divalent cations shield the repulsive electrostatic interactions between nucleosomes to compact and eventually aggregate nucleosome arrays. Despite this benefit for MAS NMR studies, Mg^2+^ presents a conundrum for sample preparation due to its profound effect on chromatin structure (Berezhnoy et al., 2016) and inconclusive results regarding its physiological concentrations in the nucleus (Maeshima et al., 2018). As a result, it is important to study chromatin in various buffer conditions and rigorously compare the outcomes. Arrays and mononucleosomes have been prepared for MAS NMR with and without Mg^2+^-induced precipitation (Gao et al., 2013; le Paige et al., 2021). Mononucleosome sediments with low levels of Mg^2+^ lacked the long-range order expected for stacked fibers formed by high Mg^2+^ concentrations (le Paige et al., 2021). Such irregular packing may illuminate the transient nucleosome-nucleosome interactions that dominate when nucleosomes are not restricted into ordered arrays (Bilokapic et al., 2018; Sanulli et al., 2019; Alvarado et al., 2021). Thus, samples prepared by sedimentation and low Mg^2+^ concentrations may prove crucial for resolving the transient interactions that lead to chromatin compaction and regulation (Gibson et al., 2019; Khanna et al., 2019; Sanulli et al., 2019; Kantidze and Razin, 2020).

## MAS NMR of Histone Tails

The flexible N- and C-terminal histone tails extend from the nucleosome, each with a distinct interaction profile and PTM landscape. The histone tails have been the subject of numerous biochemical and biophysical studies that have revealed their important role in chromatin structure, function, and regulation (Peng et al., 2021). While these dynamic segments are rarely visible on their own in X-ray and cryo-EM structural models, they can be studied by both solution and MAS NMR spectroscopy. The first MAS NMR study by Gao et al. used INEPT-based experiments to map the amino acid specific dynamics of the H3 and H4 tails within arrays at different Mg^2+^ concentrations (Gao et al., 2013). The histone tails remained dynamic regardless of the Mg^2+^ concentration used (0–5 mM), arguing for a much more flexible landscape than suggested previously by X-ray crystallography and hydrogen-deuterium exchange experiments (Luger et al., 1997; Kato et al., 2009). 2D ^1^H-^13^C and ^1^H-^15^N correlations revealed identifiable amino acids signatures that made it possible to compare the information content of INEPT-based experiments under solution and MAS NMR conditions. For example, Val 35, a unique amino acid in the H3 tail, was visible in both solution and MAS NMR spectra, while residues up to Val 21 were detected in the MAS NMR experiments of H4, in contrast to solution NMR experiments where only residues up to Ala 15 were present (Zhou et al., 2012; Morrison et al., 2021; Rabdano et al., 2021). Subsequent MAS NMR studies of mononucleosomes by Shi et al. also confirmed these observations (Shi et al., 2018). This discrepancy is most likely due to the type of experiments used (i.e., sidechain vs. backbone correlations), with ^13^C MAS NMR experiments holding a slight advantage regarding the detection of slower moving tail sidechains such as those that are close to the DNA interface (Shi et al., 2018; Shi et al., 2020b). While the detected tail boundary is consistent between arrays and mononucleosomes in MAS NMR experiments, a closer look into the ^15^N spin relaxation rates of H3 tails reveals mobility differences (Zandian et al., 2021). Here, T_1_ and T_1*ρ*_ relaxation measurements were used to quantify residue-specific rotational correlation times. The H3 tail was most dynamic in 147 bp mononucleosomes, the mobility was diminished by linker DNA extensions and was further reduced in nucleosome arrays. These results are consistent with solution NMR studies that show H3 tail dynamics to be regulated by transient DNA contacts (Stutzer et al., 2016).

Histone tail interactions with DNA can tuned by the chemical properties of specific PTMs. H4 lysine 20 trimethylation (H4 K20me3), for example, is a hydrophobic modification thought to increase the compaction of nucleosome arrays by altering the adjacent residue side-chain interactions with DNA (Lu et al., 2008). Shoaib et al. used MAS NMR to show that H4 K20 mono- and tri-methylation differentially dictate tail conformation and lead to either open or closed chromatin states, respectively (Shoaib et al., 2021). These conclusions were based on genomic accessibility studies, Mg^2+^ precipitation experiments and 2D ^1^H-^13^C INEPT correlations that focused on Val 21, a residue that is, adjacent to the modification site. Surprisingly, the Val 21 Hα-Cα correlation was split into two peaks for the non- and tri-methylated samples but only one peak was visible for the monomethylated H4 tail. While it is not yet clear how these changes in H4 tail dynamics and structure bring about open or closed chromatin states, this study exemplifies the power of MAS NMR to monitor tail dynamics in the context of nucleosome arrays where the inter-nucleosome contacts are essential to the structural hypothesis.

MAS NMR has also revealed that histone tails can sense their local environment and drive chromatin compaction while remaining dynamic and potentially accessible to regulatory proteins. A recent study by le Paige et al. confirmed that the dynamic tails sustain accessibility within dense sedimented samples (le Paige et al., 2021). In this case, the interactions of the histone reader domain PHD2 with histone H3 were compared by INEPT-based experiments in nucleosome sediments and under dilute conditions. For MAS NMR, PHD2 was co-sedimented with nucleosomes during ultracentrifugation, while the dilute samples could be analyzed by solution NMR. The fast MAS rate of 50 kHz afforded ^1^H-detection and generated highly resolved spectra of the histone tail backbone. Crucially, the spectral quality provided residue-specific assignments to directly compare the isotropic chemical shifts with solution NMR experiments. PHD2 binding produced comparable ^1^H-^15^N resonance broadening of residues 3–10 in both conditions. While the weak affinity of PHD2 complicated quantitative comparison of binding interactions, this study confirmed the permeability of dense chromatin environments to histone readers. This strategy will undoubtedly prove useful in future experiments aimed to dissect the interactions between histone readers and their nucleosome binding sites.

## MAS NMR of the Nucleosome Core

While the dynamic histone tails are a major target for modification and binding, sequence variations, PTMs and disease-related mutations also embroider the nucleosome core and can disturb fundamental processes like nucleosome sliding, histone exchange, and DNA wrapping (Bowman and Poirier, 2015; Fenley et al., 2018; Bryant et al., 2020; Bagert et al., 2021). In addition, many chromatin readers interact with the nucleosome core which provides additional interaction surfaces through the H2A/H2B acidic patch and the histone/DNA interface. Solution NMR, and in particular methyl-TROSY spectroscopy, has illuminated nucleosome core motions resulting from histone mutations (Kitevski-LeBlanc et al., 2018), DNA methylation (Abramov et al., 2020), and interacting proteins (Kato et al., 2011; Sanulli et al., 2019). However, only a few amino acid types are typically detectable due to the necessary methyl-labeling scheme. A major advantage of MAS NMR is the ability to characterize the entire nucleosome core in the same samples and conditions as those used to analyze the dynamic histone tails. The Nordenskiöld lab has pioneered MAS NMR of the nucleosome core by extensively assigning the highly resolved histone H3 and H4 spectra obtained with CP-based ^15^N-^13^C-^13^C three-dimensional experiments (Shi et al., 2018; Shi et al., 2020a). In the first of several studies, histone H4-labeled mononucleosomes and 12-mer arrays were Mg^2+^-precipitated and subjected to CANCO and quantitative DIPSHIFT experiments which capture motion on a wide ns-ms timescale (Munowitz et al., 1982; Shi et al., 2018). Motions at the ns-scale involve side-chain fluctuations and μs-ms motions correspond to larger domain movements (Lewandowski, 2013). The array and mononucleosome samples were consistent in overall structure and dynamics. Small signal intensity differences were observed for residues adjacent to the N-terminal H4 tail, suggesting that the base of the tail is more rigid in the chromatin fiber. The mononucleosome samples in this study were precipitated with 20 mM Mg^2+^ which generates columnar stacked assemblies that may dictate histone dynamics and influence interpretation (Berezhnoy et al., 2016). Nonetheless, the precedent of quantitative dynamic parameters for each histone residue created a platform for probing other histones and DNA sequences.

Histone H3 spectra soon followed, illustrating that both H3 and H4 experience some ns-μs motions and have regional clusters of moderately altered μs-ms motions and highly dynamic termini (Shi et al., 2018; Shi et al., 2020b). Together, these results suggest histone H3 and H4 form stable folds but can undergo local intermediate motions. When these motions are mapped on the nucleosome structure, small neighboring clusters of dynamic sites connect from the nucleosome core to the DNA-bound periphery. Correlative motions of residues that contact DNA may be important for regulating biological processes like DNA wrapping, sliding, and nucleosome assembly (Bowman and Poirier, 2015; Sinha et al., 2017; Sanulli et al., 2019; Bagert et al., 2021). The connection between histone and DNA dynamics was supported by comparing nucleosomes formed with the 601 sequence to those prepared with a tandem-repeat (TTAGGG) telomere sequence. The TTAGGG nucleosomes displayed a greater range of motions in the cluster network compared to the 601 nucleosomes consistent with previous experiments which showed that telomeric nucleosomes are less stable and wrap DNA less tightly (Shi et al., 2020b; Soman et al., 2020). Reduced nucleosome stability may translate into more flexible chromatin fibers that in turn enhance the potential for phase separation at telomeres (Sanulli et al., 2019; Farr et al., 2021). Here, MAS NMR greatly contributed to establishing a connection between histone core dynamics and the compaction of chromatin fibers. Further broadening the scope of DNA to include other genetic sequences and DNA modifications will allow MAS NMR to bridge the vast expanse of genomic data with nucleosome-specific dynamics.

MAS NMR has also been used to detect interactions between the nucleosome core and regulatory proteins. The Baldus and van Ingen labs have demonstrated the sensitivity of MAS NMR to chemical environment changes when the H2A/H2B acidic patch is bound by a peptide segment of the viral LANA protein (Xiang et al., 2018). In this study, LANA and mononucleosomes were co-sedimented with 2 mM Mg^2+^. ^1^H-detected CP experiments were used to assign 93% of the H2A core backbone, locate the LANA binding site, and independently model the binding conformation in agreement to the crystal structure. ^1^H-detection was crucial to observe significant chemical shift perturbations (CSPs) that were diminished in the ^13^C and ^15^N dimensions. Importantly, the absence of peak-splitting suggests that the LANA peptide bound both sides of the nucleosome simultaneously. Such an observation is important because the LANA peptide affects chromatin compaction by shielding the acidic patch (Chodaparambil et al., 2007). Detection of acidic patch interactions by ^1^H-detected MAS NMR holds promise for studying the myriad of chromatin modulators that use basic disordered segments to bind the nucleosome (Mashtalir et al., 2020; Teles et al., 2020).

## MAS NMR of Chromatin Modulators

Almost all MAS NMR studies of chromatin so far have focused on the nucleosome perspective, while the structure, dynamics, and interactions of chromatin modulators have largely remained unexplored. Here, MAS NMR can offer unique advantages as often chromatin modulators are too large to study by solution NMR or they form dynamic, viscous and heterogenous phase separated states that cannot be dissected by single particle techniques such as cryo-EM. Most importantly, however, such studies can be performed in the presence of mononucleosomes or nucleosome arrays where isotopic labeling of each protein one at a time can provide an opportunity to analyze a single component at high resolution in the complex chromatin-modulator assembly. A study from our lab used MAS NMR to illuminate the liquid-liquid phase separation of phosphorylated heterochromatin protein 1α (pHP1α) in the presence and absence of nucleosome arrays (Ackermann and Debelouchina, 2019). HP1α is a key chromatin modulator responsible for the formation of heterochromatin domains in the nucleus where silenced genes are sequestered (Cheutin, 2003). N-terminal serine phosphorylation was previously found to enhance the phase separation behavior of HP1α and to promote the transition from dynamic liquid to an arrested gel state (Larson et al., 2017). To measure the phenomenon in a more physiological chromatin environment, phase separated and isotopically labeled pHP1α was packaged into MAS rotors with and without H3 lysine 9 trimethylated nucleosome arrays, the native binding partner for pHP1α. Both INEPT and CP experiments were employed to measure the sample dynamics during gelation. The addition of chromatin appeared to disrupt the pHP1α-pHP1α gel network as detected by the lower overall CP signal for pHP1α. In addition, 2D INEPT-TOBSY and CP-based DARR ^13^C-^13^C correlations revealed changes in the dynamics of specific serine residues as pHP1α transitioned from the liquid to the gel state. In this case, MAS NMR provided an opportunity to observe in real time molecular interactions that underlie the formation of phase separated chromatin environments. As many chromatin modulators are now known to undergo phase separation (Weber, 2019), these unique capabilities of MAS NMR can no doubt be exploited further to provide a much needed molecular view of this complex biological process.

## Chemical Biology Tools for MAS NMR of Modified Chromatin Samples

So far, MAS NMR studies of chromatin have largely used samples prepared with recombinant histones that are devoid of PTMs. Considering the importance of histone modifications in defining chromatin structure, dynamics and function, it is necessary to consider efficient and specific modification strategies that are compatible with isotopic labeling. In some serendipitous cases, enzymatic modification during expression in *E. coli* or after purification may produce homogeneously modified proteins. For example, we have used co-expression with the kinase CK2 to prepare isotopically labeled and well-defined phosphorylated HP1α samples with high yields (Ackermann and Debelouchina, 2019). More often, however, enzymatic approaches result in incomplete or off target modifications. Therefore, chemical approaches that introduce PTMs in a well-defined and controlled manner are highly desirable (Debelouchina and Muir, 2017).

**FIGURE 3:**
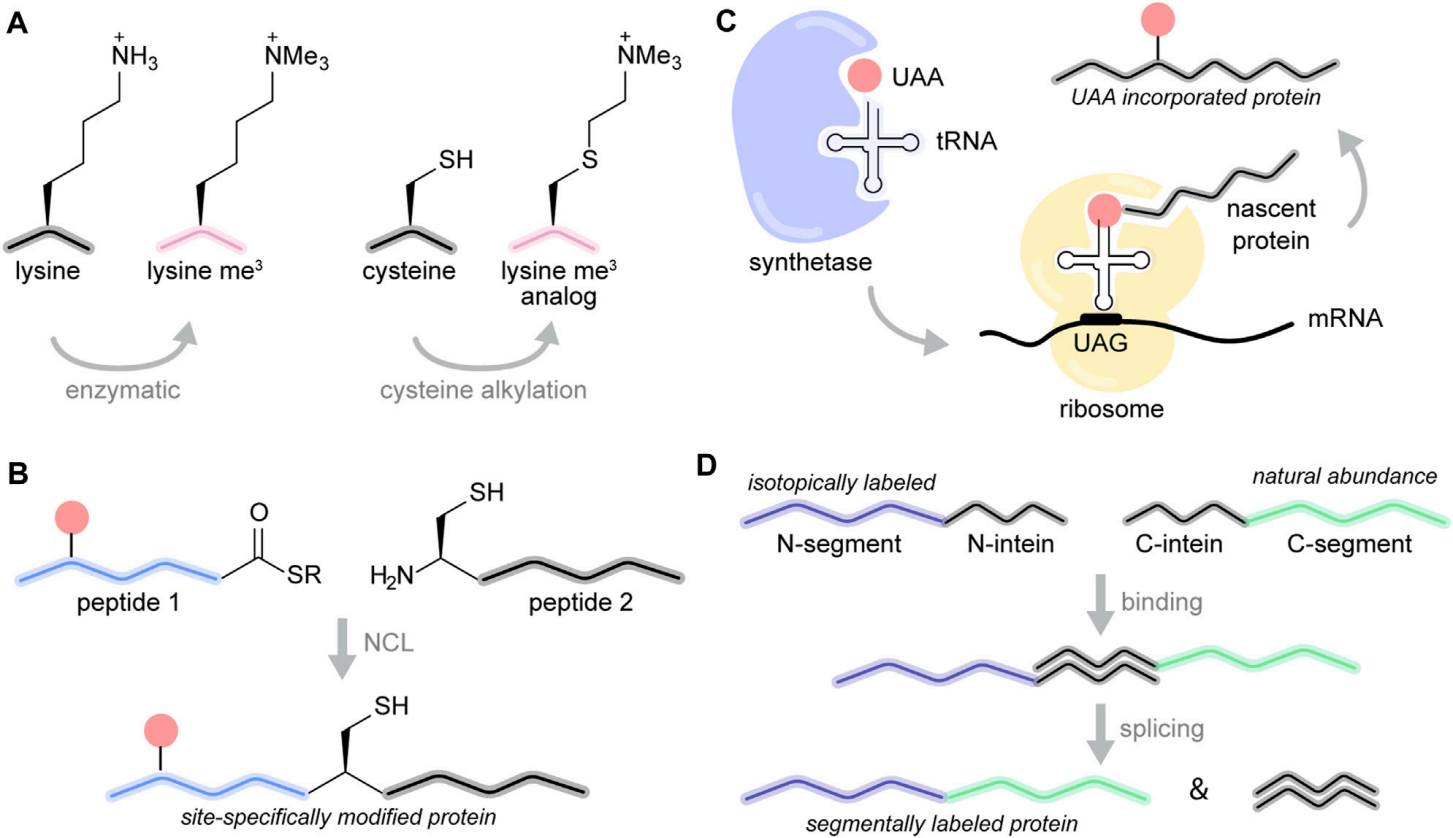
Chemical biology toolbox for chromatin studies. (**A**) Lysine trimethylation (me^3^) can be installed enzymatically or by cysteine alkylation to yield a methylated lysine analog. (**B**) In native chemical ligation, a synthetic peptide containing a C-terminal thioester (1) is linked to a second polypeptide bearing an N-terminal cysteine (2). (**C**) In unnatural amino acid incorporation, the UAA is loaded onto the corresponding tRNA by an engineered tRNA synthetase. The tRNA recognizes the amber stop codon UAG, allowing the ribosome to install the UAA at the desired position in the protein sequence. (**D**) Segmental isotopic labeling is mediated by intein splicing of an isotopically labeled protein segment with a segment at natural abundance, producing the full-length protein.

Chromatin templates have long served as an expansive and challenging canvas for the development of chemical biology methods for protein modification (Allis and Muir, 2011). The histone proteins are relatively small (ranging in size from 100 to 150 amino acids) and practically devoid of cysteine residues (the lone Cys110 on H3.2 can easily be replaced with an alanine or serine residue without loss of structure or function). This makes the application of cysteine-based modification approaches relatively straightforward (Chalker et al., 2009; Boutureira and Bernardes, 2015). Cysteine modification, for example, has been widely used to introduce spectroscopic probes, including fluorescent labels and paramagnetic relaxation enhancement (PRE) tags. More importantly, however, cysteine alkylation provides a convenient and efficient strategy to prepare lysine methylation mimics (Simon et al., 2007). In this case, the targeted lysine residue is replaced with a cysteine, and the cysteine is modified with mono-, di-, or trimethyl containing alkylating reagents (Figure 3A). The reaction is usually performed with the purified protein under denaturing conditions and is compatible with isotopic labeling provided that the protein can be refolded. While the resulting mimic contains a sulfur instead of a carbon atom at the *γ* position of the side chain, *in vitro* biochemical studies have shown that this mimic can faithfully reproduce the functional consequences of lysine methylation (Simon et al., 2007). We have successfully used this strategy to prepare large amounts of nucleosome array samples that contained H3 K9me3 (Ackermann and Debelouchina, 2019), the relevant modification for HP1α binding and heterochromatin formation. This strategy was also used to explore the effects of K20 methylation on the dynamics of the H4 tail (Shoaib et al., 2021).

Multiple PTMs can be introduced with a technique called native chemical ligation (NCL) (Dawson and Kent, 2000). In this case, the N-terminal segment of the protein (typically the first 10 to 50 residues) is made by solid-phase peptide synthesis and post-translationally modified amino acids can be introduced at specific positions in the sequence as desired (Figure 3B). The peptide ends with a C-terminal thioester necessary for the subsequent ligation step. At the same time, the remaining C-terminal segment of the protein can be prepared recombinantly in *E.coli* with or without isotopic labeling (Muir et al., 1998). This segment requires an N-terminal cysteine for ligation. Upon mixing, the synthetic peptide and the recombinant piece undergo a set of thio-esterification steps that result in a native peptide bond at the ligation junction. If necessary, the cysteine residue at the junction can be converted to an alanine by desulfurization (Yan and Dawson, 2001). It is also possible to adapt this technique to perform three-piece ligations and to introduce modifications in the middle or the C-terminal segment of the histone protein (Hackeng et al., 1999). The unprecedented chemical control and versatility of NCL has been used to construct large libraries (with more than 100 members) of uniquely modified nucleosomes and to elucidate the mechanisms of chromatin remodeling and multivalent PTM readout (Nguyen et al., 2014; Dann et al., 2017). While NCL has not yet been applied for MAS NMR of chromatin, it has been impactful in numerous solution and MAS NMR studies of other proteins (Kwon et al., 2015; Zoukimian et al., 2019).

Bypassing the need for cysteines and synthesis, single PTMs can be introduced into proteins using unnatural amino acid (UAA) incorporation by genetic means (amber suppression) (Lang and Chin, 2014) (Figure 3C). Typically, the amber stop codon (TAG/UAG) is used to signal the position where the unnatural amino acid will be placed. To interpret this message correctly, the ribosome requires an engineered tRNA that can recognize this codon and is loaded with the UAA. The tRNA and the gene for an appropriately engineered matching tRNA synthetase that loads the UAA onto the tRNA are typically encoded onto a separate plasmid. *E. coli* cultures are then transformed with the tRNA/tRNA synthetase plasmid and a plasmid that contains the gene for the protein of interest with the TAG mutation. The unnatural amino acid is added to the media, and expression and purification of the modified protein can be performed as usual. While amber suppression systems have been engineered for the introduction of more than 100 UAAs (Liu and Schultz, 2010), the UAAs of particular interest to the chromatin structural biologist are acetylated lysine, phosphorylated serine and UAA precursors that can be converted into methylated amino acids (Neumann et al., 2008; Pirman et al., 2015; Wang and Liu, 2017). The major drawback of amber suppression for MAS NMR studies of chromatin is that it results in much lower yields of the desired protein, a situation that can be severely exacerbated under isotopic labeling conditions. Nevertheless, we have successfully used this technique to introduce UAAs in different isotopically labeled proteins, including some that are relevant for chromatin studies (Lim et al., 2020).

Finally, it is important to mention intein-mediated segmental labeling (Muir et al., 1998; Shah and Muir, 2014), a useful tool for proteins with congested NMR spectra (Züger and Iwai, 2005; Schubeis et al., 2015; Frederick et al., 2017; Gupta and Tycko, 2018; Wiegand et al., 2018; Ciragan et al., 2020) (Figure 3D). Split inteins are a class of proteins found in unicellular organisms that can “stitch” together other protein segments with a native peptide bond. To prepare segmentally labeled samples for NMR spectroscopy, the desired protein is split into two segments. The N-terminal segment is attached to an N-terminal split intein while the C-terminal segment is attached to the matching C-terminal intein piece. The N- and C-segments can be prepared independently in different cultures, for example, one using ^13^C, ^15^N labeling and the other at natural abundance. Once the segments are purified, mixing of the segments results in an interaction between the intein pieces which adopt a functional intein fold. The intein mediates the formation of a native peptide bond between the two protein segments while excising itself in the process. This process requires a cysteine residue at the ligation junction. The result is a full-length protein with only a segment of the sequence visible by NMR, thereby simplifying the acquired spectra. This can aid assignment protocols (Iwai and Züger, 2007) and provide the opportunity to probe specific inter- or intra-molecular interactions (Frederick et al., 2017). There is now a large variety of efficient split intein pairs that can be used for this purpose (Pinto et al., 2020), including some that work well under denaturing conditions (Stevens et al., 2016). We envision that this technique will be extremely valuable in the resonance assignment and MAS NMR analysis of chromatin interacting proteins.

## Discussion

While still few in number, the recent applications of MAS NMR have demonstrated its versatile capabilities in the structural analysis of chromatin samples. Uniquely capable of characterizing both the rigid and dynamic components of mononucleosome and array preparations, precipitated, sedimented, and concentrated samples, chromatin MAS NMR has illuminated fundamental aspects of nucleosome dynamics, histone tail recognition, acidic patch docking, and heterochromatin phase separation. Most chromatin experiments so far have relied on dipolar and scalar based experiments performed at moderate spinning frequencies (10–20 kHz) and the detection and analysis of ^13^C and ^15^N signals. We expect that ^1^H-detection and fast MAS (at 100 kHz and beyond) will continue to improve signal sensitivity and facilitate the resolution of chemical shift perturbations and chromatin interactions (Andreas et al., 2016; Struppe et al., 2017). Since chromatin assemblies contain multiple proteins and DNA, the preparation of large amounts of isotopically labeled samples can be time consuming and challenging, especially if chromatin interacting proteins or PTMs are included. We therefore expect that such samples will benefit tremendously from sensitivity-enhancement developments such as dynamic nuclear polarization and cryo-MAS probes (Lilly Thankamony et al., 2017; Hassan et al., 2020). Chemical biology tools such as cysteine alkylation, native chemical ligation and amber suppression enable the preparation of specifically decorated chromatin, while inteins allow the simplification of crowded histone or chromatin reader spectra. We envision that the combination of chemical biology tools and MAS NMR will provide the unprecedented opportunity to construct and dissect complex chromatin environments where dynamic multifaceted interactions can be interrogated one at a time.
